# Investigating the role of *MAB_1915* in intrinsic resistance to multiple drugs in *Mycobacterium abscessus*

**DOI:** 10.1128/spectrum.03974-23

**Published:** 2024-08-20

**Authors:** Buhari Yusuf, Shuai Wang, Md Shah Alam, Jingran Zhang, Zhiyong Liu, Ziwen Lu, Jie Ding, Gift Chiwala, Yamin Gao, Cuiting Fang, Shahzad Akbar Khan, Xirong Tian, Md Mahmudul Islam, H. M. Adnan Hameed, Dmitry A. Maslov, Nanshan Zhong, Jinxing Hu, Tianyu Zhang

**Affiliations:** 1 State Key Laboratory of Respiratory Disease, Guangzhou Institutes of Biomedicine and Health, Chinese Academy of Sciences, Guangzhou, China; 2 Guangdong-Hong Kong-Macao Joint Laboratory of Respiratory Infectious Diseases, Guangzhou, China; 3 China-New Zealand Joint Laboratory on Biomedicine and Health, Guangzhou Institutes of Biomedicine and Health, Chinese Academy of Sciences, Guangzhou, China; 4 University of Chinese Academy of Sciences, Beijing, China; 5 School of Life Sciences, University of Science and Technology of China, Hefei, China; 6 Guangzhou Medical University, Guangzhou, China; 7 Guangzhou National Laboratory, Guangzhou, China; 8 Institutes of Physical Science and Information Technology, Anhui University, Hefei, China; 9 Malawi Liverpool Wellcome Clinical Research Programme, Blantyre, Malawi; 10 Laboratory of Pathology, Department of Pathobiology, University of Poonch Rawalakot Azad Kashmir, Rawalakot, Pakistan; 11 Division of Gastroenterology and Hepatology, Department of Medicine, Stanford University School of Medicine, Stanford, California, USA; 12 State Key Laboratory of Respiratory Disease, National Clinical Research Center for Respiratory Disease, The National Center for Respiratory Medicine, The First Affiliated Hospital of Guangzhou Medical University, Guangzhou, China; 13 State Key Laboratory of Respiratory Disease, Guangzhou Chest Hospital, Guangzhou, China; Johns Hopkins University School of Medicine, Baltimore, Maryland, USA

**Keywords:** *Mycobacterium abscessus*, MAB_1915, fatty acid-CoA ligase, cell envelope permeability, intrinsic drug resistance

## Abstract

The increasing clinical significance of *Mycobacterium abscessus* is owed to its innate high-level, broad-spectrum resistance to antibiotics and therefore rapidly evolves as an important human pathogen. This warrants the identification of novel targets for aiding the discovery of new drugs or drug combinations to treat *M. abscessus* infections. This study is inspired by the drug-hypersensitive profile of a mutant *M. abscessus* (U14) with transposon insertion in *MAB_1915*. We validated the role of *MAB_1915* in intrinsic drug resistance in *M. abscessus* by constructing a selectable marker-free in-frame deletion in *MAB_1915* and complementing the mutant with the same or extended version of the gene and then followed by drug susceptibility testing. Judging by the putative function of MAB_1915, cell envelope permeability was studied by ethidium bromide accumulation assay and susceptibility testing against dyes and detergents. In this study, we established genetic evidence of the role of *MAB_1915* in intrinsic resistance to rifampicin, rifabutin, linezolid, clarithromycin, vancomycin, and bedaquiline. Disruption of *MAB_1915* has also been observed to cause a significant increase in cell envelope permeability in *M. abscessus*. Restoration of resistance is observed to depend on at least 27 base pairs upstream of the coding DNA sequence of *MAB_1915*. MAB_1915 could therefore be associated with cell envelope permeability, and hence its role in intrinsic resistance to multiple drugs in *M. abscessus*, which presents it as a novel target for future development of effective antimicrobials to overcome intrinsic drug resistance in *M. abscessus*.

**IMPORTANCE:**

This study reports the role of a putative fadD (MAB_1915) in innate resistance to multiple drugs by *M. abscessus*, hence identifying MAB_1915 as a valuable target and providing a baseline for further mechanistic studies and development of effective antimicrobials to check the high level of intrinsic resistance in this pathogen.

## INTRODUCTION

Mycobacteria can be fast- or slow-growing. The slow growers include members of the *Mycobacterium tuberculosis* complex. *Mycobacterium abscessus* belongs to the fast-growing group and is an opportunistic pathogen that poses a serious public health threat, particularly in the presence of underlying host factors such as cystic fibrosis, bronchiectasis, compromised immunity, and co-infection with other bacteria (1). Lipid metabolic genes such as fatty acid-CoA ligases (FACLs/FadDs) are important to mycobacteria in virulence and successful establishment of infection in their hosts (1–6). The importance of FadDs to mycobacteria could be deduced from their sheer numbers in these bacteria. For example, the genomes of each of *M. abscessus* and *M. smegmatis* encode no less than 30 FadDs, whereas that of *M. tuberculosis* encodes 36 (2). This therefore entails possible redundancies or varying roles of these genes (2, 7, 8), which indicates their importance in mycobacterial lipid metabolism.

*M. abscessus* is a non-tuberculous species that is rapidly evolving as an important human pathogen. Its public health significance cannot be ignored due to its innate high-level, broad-spectrum resistance to available antibiotics (9). This is owed to a plethora of innate, adaptive, and acquired genetic mechanisms of drug resistance in this pathogen (1, 6), including intrinsic impermeability of the mycobacterial cell envelope (1, 10). This is because the mycobacterial cell envelope is heavily guarded by a collection of lipids and mycolic acids (11–15), the biosynthesis of which is influenced by FadDs (6, 16). The apparent importance of lipid metabolic genes warrants investigation to explore whether they could be presented as promising targets in future development of antimicrobials to address the intrinsic high-level, broad-spectrum resistance to drugs by *M. abscessus*.

This study was inspired by the drug sensitivity profile of a mutant *M. abscessus* strain with transposon (Tn) insertion in a putative *fadD* (*MAB_1915*) that appears to show increased sensitivity to rifampicin. We further established the genetic evidence of the role of this gene, which has previously been implicated in α’-mycolate biosynthesis in *M. abscessus* ATCC 19977 (6), in intrinsic resistance to multiple drugs. We therefore present *MAB_1915* as a novel target for further studies and future development of effective chemotherapeutic regimens against *M. abscessus* infections.

## RESULTS

### *MAB_1915* mediates drug resistance in *M. abscessus*

**Fig 1:**
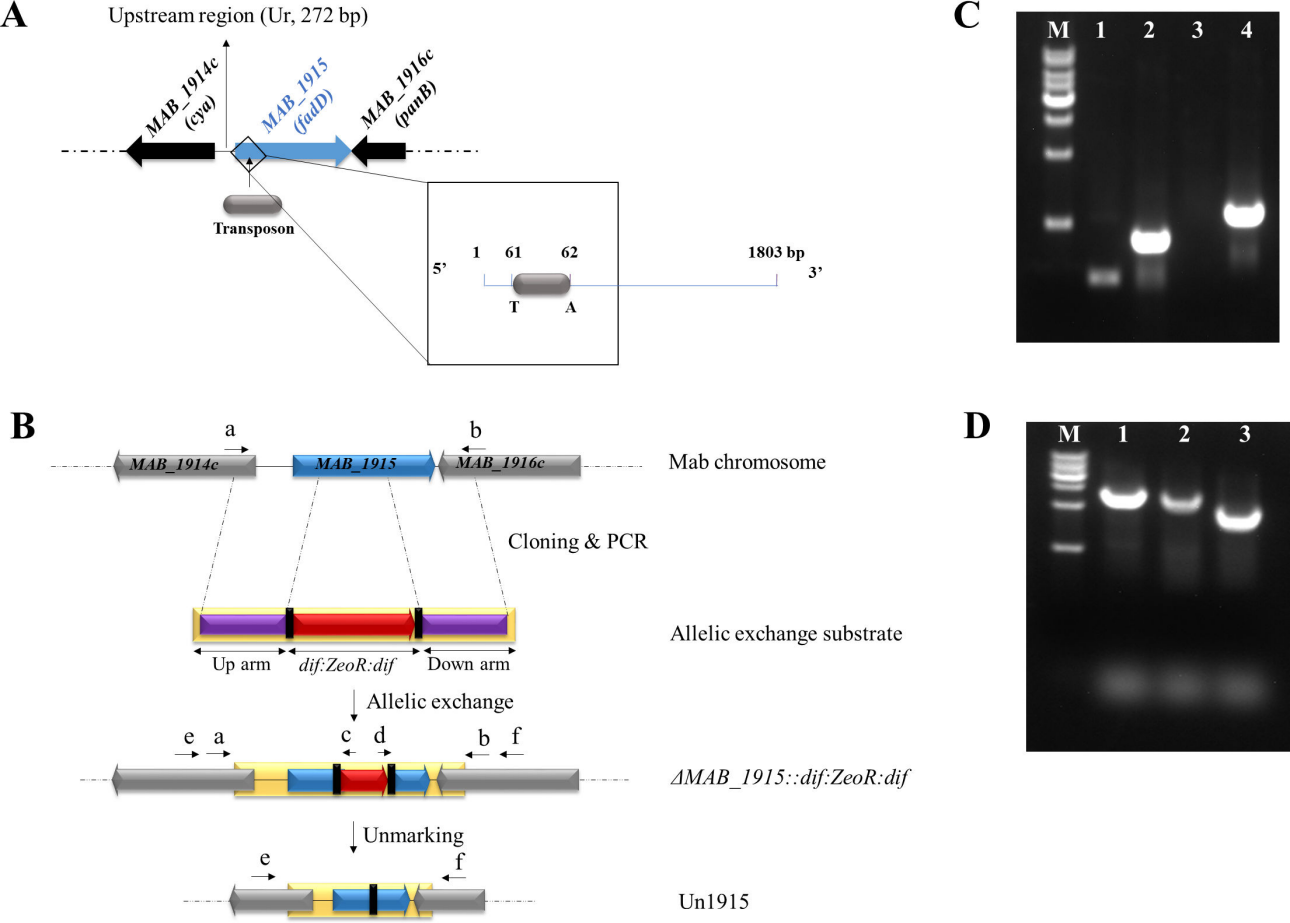
Tn insertion and construction of an in-frame disruption in *MAB_1915*. A Position of Tn insertion in U14, a Tn insertion mutant in which the Tn is seen to integrate into *M. abscessus* chromosome between “T” and “A” dinucleotides at positions 61 and 62 within *MAB_1915,* respectively. This study was inspired by the drug-sensitivity profile of this mutant. The 272-bp intergenic region between *MAB_1915* and *MAB_1914*c is also shown. B Depiction of the construction of AES, in-frame disruption of *MAB_1915* by allelic exchange and unmarking of the gene disruption mutant. C Verification of gene disruption by amplifying the junctions between the chromosome of *M. abscessus* and the inserted *zeoR* cassette. Lane M, 1-kb DNA ladder; lanes 1 and 2, Mab^Wt^ and *∆MAB_1915* mutant, respectively (primers a/c, 726 bp); lanes 2 and 4, Mab^Wt^ and *∆MAB_1915* mutant, respectively (primers d/b, 1154 bp). D Verification of knockout by amplifying (primers e/f) disrupted *MAB_1915* in Mab^Wt^, *∆MAB_1915* mutant, and unmarked *∆MAB_1915* mutant (Un1915). Lane M, 1-kb DNA ladder; lane 1, Mab^Wt^ (2672 bp); lane 2, *∆MAB_1915* mutant (2564 bp); lane 3, Un1915 (1964 bp).

The mutant *M. abscessus* U14 sustained a Tn insertion in *MAB_1915* (Fig. 1A), which encodes a putative FadD. We have reported the details of Tn mutagenesis and library screening elsewhere (17). In the present study, we carried out further genetic evaluation to investigate the possible role of *MAB_1915* in intrinsic drug resistance in *M. abscessus*.

An in-frame deletion mutant of *M. abscessus* for *MAB_1915 (∆MAB_1915*) was generated using an allelic exchange substrate (AES) comprising a Zeocin (ZEO) resistance cassette (with *dif* sites at both ends) sandwiched between upstream and downstream arms of *MAB_1915*, with both arms containing flanking sequences from adjacent genes (Fig. 1B). Gene disruption was verified by polymerase chain reaction (PCR) and sequencing, which reveals that a 708-bp fragment within *MAB_1915* was replaced by the ZEO resistance cassette (Fig. 1B). This was further verified by PCR amplification of junctions between the ZEO resistance cassette and *M. abscessus* chromosome (Fig. 1B and C). PCR products from the *∆MAB_1915* mutant have bands at both junctions, which were absent in Mab^Wt^ (Fig. 1C), further confirming successful disruption of *MAB_1915*. The ZEO resistance cassette was excised prior to downstream studies using the Xer/*dif* excision system (18) to minimize possible polar effects from the ZEO resistance marker on the expression of downstream genes on the *M. abscessus* chromosome. The resulting strain was verified by PCR and sequencing (Fig. 1B and D), then designated unmarked *∆MAB_1915* mutant (Un1915), and used as the background strain for construction of complemented (CP) strains.

**Fig 2:**
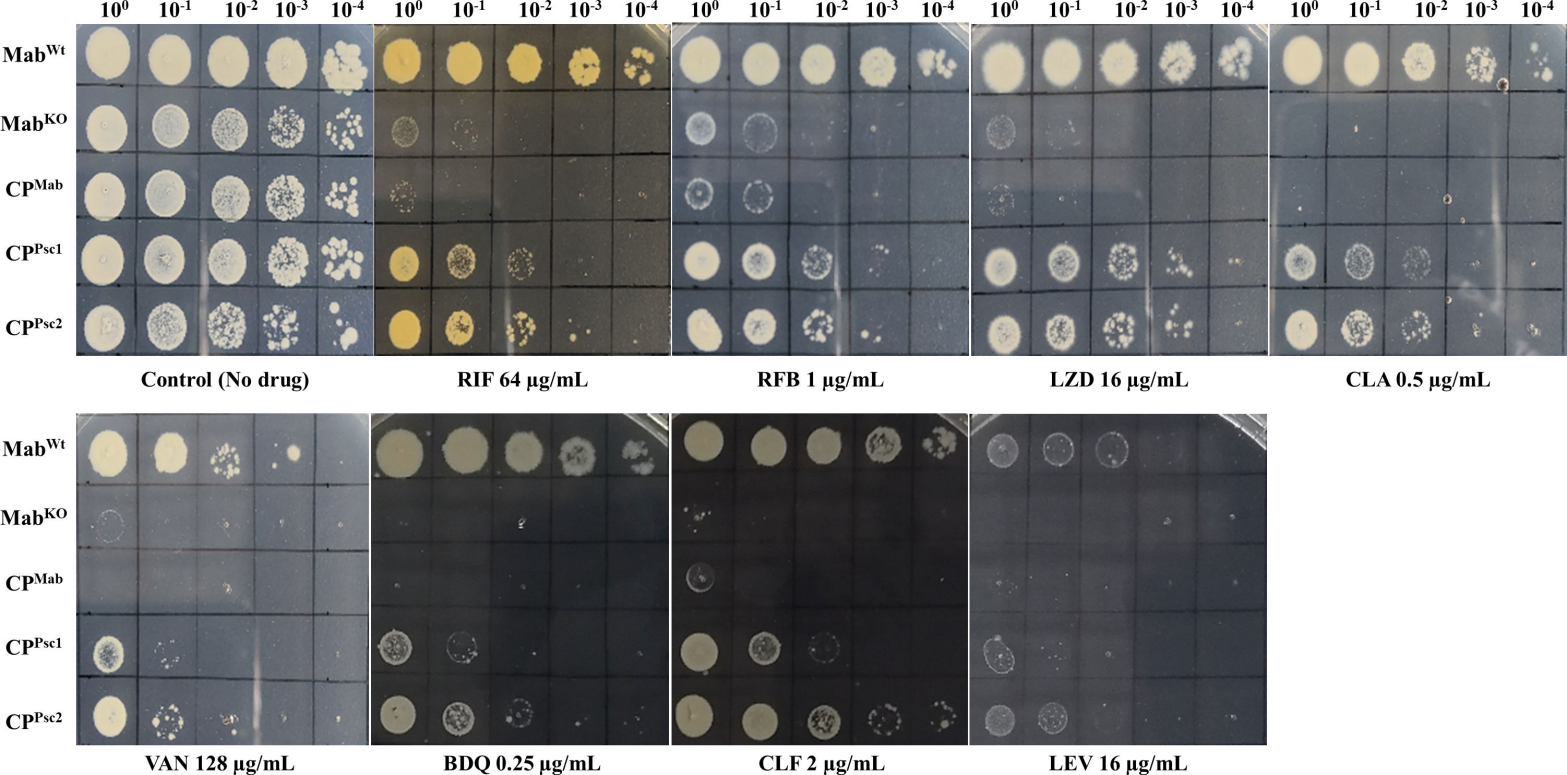
Drug susceptibility testing of the strains by SPOTi. Mab^KO^ is susceptible to RIF, RFB, LZD, CLA, VAN, and BDQ by both broth microdilution and SPOTi. However, it is observed to have some levels of susceptibility to CLF and LEV only on agar plates. The shown CP strains appear to partially restore resistance to the drugs.

The drug susceptibility profile of the *∆MAB_1915* mutant was checked against rifampicin to validate the phenotype of the Tn insertion mutant. Drug susceptibility testing (DST) reveals that *MAB_1915* disruption sensitizes *M. abscessus* to not only RIF but also to linezolid (LZD), vancomycin (VAN), rifabutin (RFB), clarithromycin (CLA), and bedaquiline (BDQ) when screened by broth microdilution (Table 1). In addition, the strain has also been observed to show some level of susceptibility to clofazimine (CLF) and levofloxacin (LEV) when screened by spot culture growth inhibition (SPOTi) (Fig. 2). Together, this demonstrates the significant impact of *MAB_1915* disruption on the drug susceptibility profile of *M. abscessus*. To verify whether the gene is indeed responsible for resistance to those drugs, the coding DNA sequence (CDS) of *MAB_1915* was expressed in Un1915 to construct the CP strain CP^Mab^ to check for restoration of the resistance phenotype by broth microdilution. The minimum inhibitory concentrations (MICs) of all the drugs were consistently lower against Mab^KO^ than those against Mab^Wt^ and remained unchanged after complementation (data not shown).

**TABLE 1:** Drug susceptibility testing of strains by broth microdilution

Strains	Characteristics of the strains^*a*^	Drugs/MIC (µg/mL)^*b*^												
		RIF	LZD	RFB	CLA	VAN	BDQ	CLF^*c*^	LEV^*c*^	IMP	EMB	CFX	TGC	AMK
Mab^Wt^	The wild-type *M. abscessus*	128	64	8	4	128	4	4	32	128	128	32	8	16
Mab^KO^	Un1915::pMV261	16	4	1	1	8	0.5	4	16	128	128	32	8	16
CP^Mab^	Un1915::*hsp60-MAB_1915*	16	16	2	1	8	0.5	−	−	−	−	−	−	−
CP^Psc1^	Un1915::*hsp60-Psc1-MAB_1915*	128	64	8	4	32	4	4	16	−	−	−	−	−
CP^Psc2^	Un1915::*hsp60-Psc2-MAB_1915*	128	64	8	4	64	4	4	16	−	−	−	−	−

^*a*^ Un1915, unmarked *MAB_1915* knockout strain. pMV261, the empty extra-chromosomal plasmid. *hsp60*, the strong mycobacterial promoter. *Psc,* presumed start codon. *Psc1* and *Psc2* contain 69 and 27 bp from the upstream region (272 bp) of *MAB_1915* (Fig. 3), respectively.

^*b*^ -, not determined (because Mab^KO^ was not hyper-susceptible to these drugs); RIF, rifampicin; LZD, linezolid; RFB, rifabutin; CLA, clarithromycin; VAN, vancomycin; BDQ, bedaquiline; CLF, clofazimine; LEV, levofloxacin; IMP, imipenem; EMB, ethambutol; CFX, cefoxitin; TGC, tigecycline; AMK, amikacin.

^*c*^ Mab^KO^ was more susceptible to CLF and LEV on the agar plate (Fig. 2).

### Role of *MAB_1915* in drug resistance depends on its upstream 272-bp region

Because CP^Mab^ could not restore resistance to the tested drugs, we explored whether the upstream 272-bp region (Fig. 1A) could be a player in the role of *MAB_1915* in drug resistance. Therefore, we constructed another CP strain, CP^UrMab^, by expressing *MAB_1915* together with its upstream 272-bp region in Un1915. Interestingly, this resulted in a remarkable restoration of the drug resistance phenotype (data not shown), suggesting that the role of *MAB_1915* in resistance to the tested drugs could be dependent on whole or part of the 272-bp intergenic region between *MAB_1914*c and *MAB_1915*.

There were insignificant changes in MICs of the tested drugs following complementation of the mutant with the CDS of *MAB_1915*, demonstrating inadequate restoration of the drug resistance phenotype. However, when *MAB_1915* was extended to include its upstream 272-bp region, there was an eightfold increase in the MICs of RIF, LZD, and VAN and a fourfold increase in the case of RFB and CLA (data not shown). This therefore indicates restoration of the drug resistance phenotype by CP^UrMab^, which expresses a version of *MAB_1915* in which the CDS has been extended to include its upstream 272-bp region.

### Partial extension of CDS of *MAB_1915* into the 272-bp region

**Fig 3:**
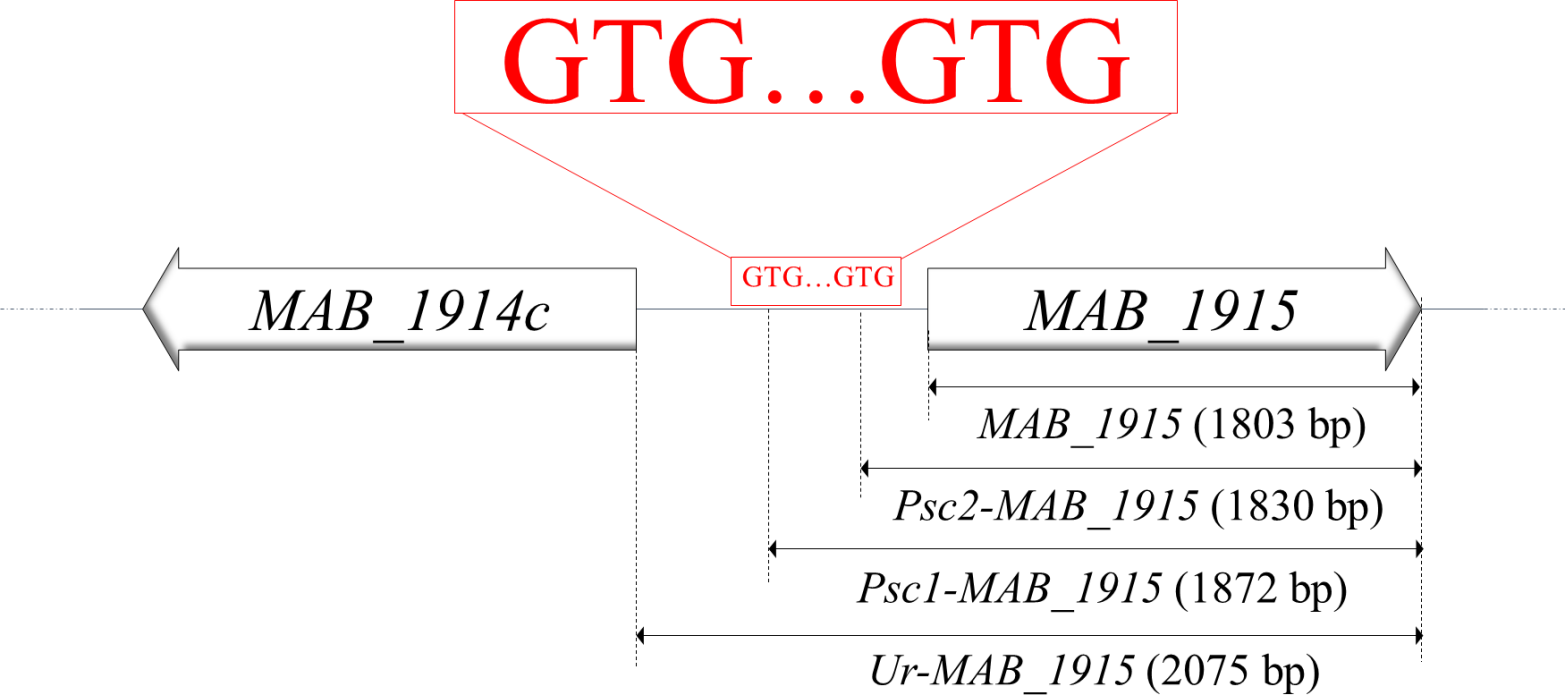
Presumed start codons (Psc) upstream of *MAB_1915*. Two alternative “GTG” start positions were presumed some 69 and 27 bp upstream of *MAB_1915*. In contrast to *MAB_1915,* the expression of either *Psc1-MAB_1915* or *Psc2-MAB_1915* in the knockout strain enables partial restoration of drug resistance.

**Fig 4:**
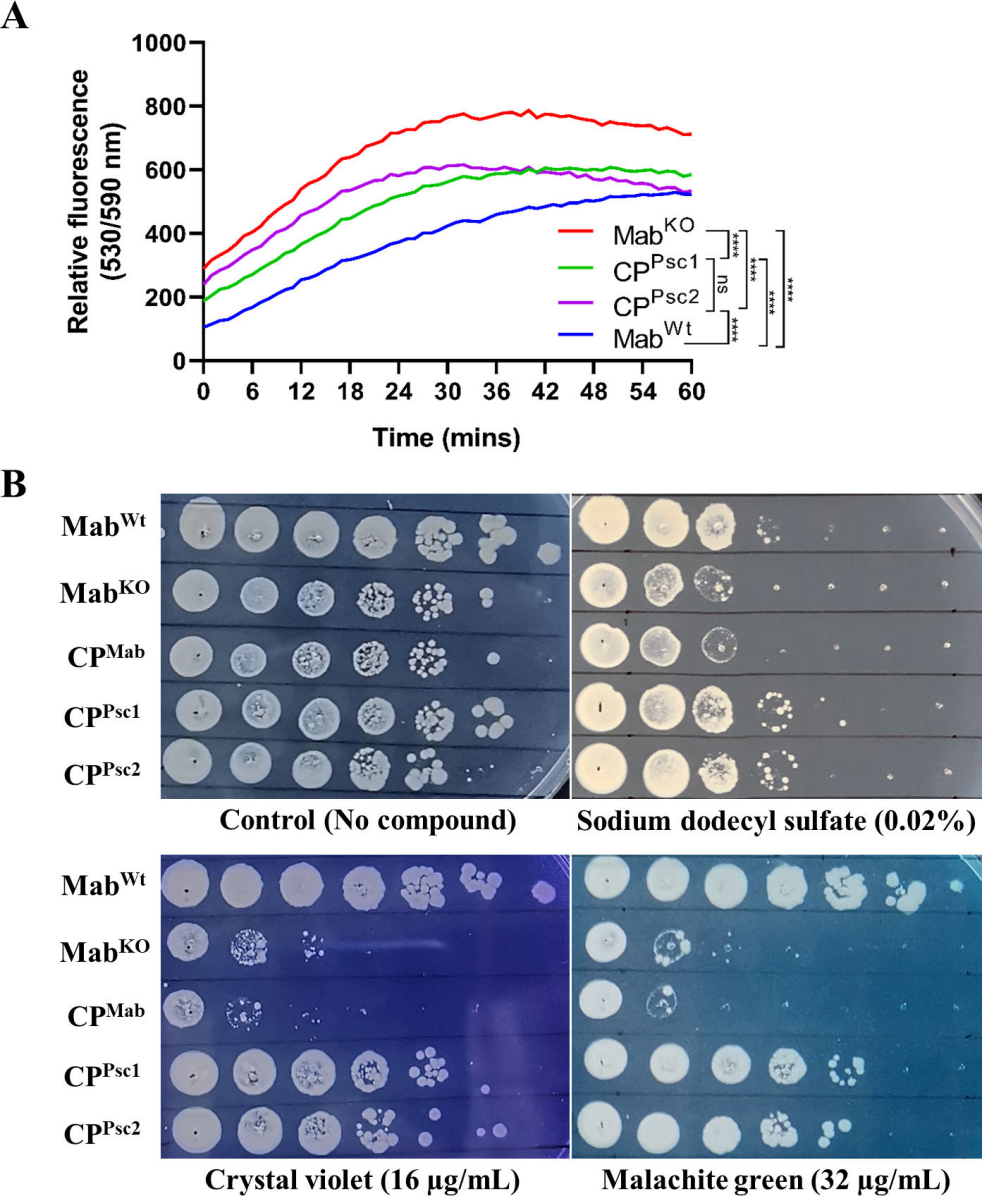
Assessment of cell envelope permeability. A Accumulation of EtBr in the strains. B Susceptibility testing against dyes and detergents. Mab^KO^ accumulated more EtBr and was also observed to be relatively more sensitive to dyes and detergents in contrast to the CP strains, which perhaps results from an increase in the permeability of its cell envelope. ^****^, *P* < 0.0001; ns, not significant.

Because extension of *MAB_1915* to include its upstream 272-bp region (*Ur-MAB_1915*) enables the mutant strain to restore resistance to drugs, sodium dodecyl sulfate (SDS), malachite green, and crystal violet (Fig. 3 and 4B), we checked whether shortening *Ur-MAB_1915* would also exert a similar effect. Thus, we identified two close, potentially alternative “GTG” start positions (19) near the original annotated start codon (ATG) of *MAB_1915*, which we designated presumed start codons 1 and 2 (*Psc1* and *Psc2*) such that *Ur-MAB_1915* §amp;gt; *Psc1-MAB_1915* §amp;gt; *Psc2-MAB_1915* §amp;gt; *MAB_1915* in length (Fig. 3). Both *Psc1-MAB_1915* and *Psc2-MAB_1915* begin at “GTG” positions some 69 and 27 bp upstream of the annotated “ATG” start codon of *MAB_1915*, respectively.

We therefore constructed two other complemented strains CP^Psc1^ and CP^Psc2^ expressing *Psc1-MAB_1915* (1872 bp) and *Psc2-MAB_1915* (1830 bp), respectively. We compared the drug susceptibility profiles of these strains to those of Mab^Wt^, Mab^KO,^ and CP^Mab^ and, interestingly, both strains restored resistance to all the tested drugs, as opposed to CP^Mab^ (Table 1; Fig. 2).

### Drug sensitivity of Mab^KO^ could be due to increased cell envelope permeability

MAB_1915 is functionally annotated as a putative FadD, and we could therefore expect it to play an important role in lipid metabolism and perhaps cell envelope permeability. To study the possible role of this protein in determining cell envelope permeability in *M. abscessus*, we carried out ethidium bromide (EtBr) accumulation assay. As expected, there was an apparent increase in EtBr uptake following *MAB_1915* disruption, a phenotype that was partially restored following complementation. Mab^KO^ showed a significantly higher rate of EtBr accumulation in contrast to Mab^Wt^ and the two CP strains CP^Psc1^ and CP^Psc2^ (*P* < 0.05), suggesting a possible increase in cell envelope permeability in the knockout strain (Fig. 4A).

Susceptibility of the strains to SDS as well as malachite green and crystal violet was determined by SPOTi, in which Mab^KO^ was observed to be apparently more susceptible than Mab^Wt^, suggesting a possible increase in cell envelope permeability. Interestingly, CP^Psc1^ and CP^Psc2^ partially restored the lost phenotype (Fig. 4B). This suggests that just like in drug resistance, the association of *MAB_1915* with cell envelope permeability could be dependent on its upstream region.

## DISCUSSION

*M. abscessus* is a rapidly emerging human pathogen, and its infection is largely influenced by the presence of underlying host factors (1). This species stands out among other non-tuberculous mycobacteria for its intrinsic high level of resistance to multiple drugs (9). This certainly warrants the understanding of the genetic basis of inherent drug resistance in this pathogen.

FadDs are adenylate-forming enzymes that play key roles in mycobacterial lipid metabolism and cell envelope biogenesis. Like *M. tuberculosis* (2), the genomes of *M. abscessus* and other mycobacteria encode multiple FadDs, some of which have been shown to mediate intrinsic resistance to antibiotics (5, 6). In this study, we identified genes related to drug resistance in *M. abscessus* by Tn mutagenesis (20, 21) and DST. A mutant, U14, from the Tn mutant library was determined to have sustained a Tn insertion in *MAB_1915* (Fig. 1A) and showed increased susceptibility to RIF.

*MAB_1915* is one of the over 30 genes that encode putative FadDs in *M. abscessus*. Here, we established a genetic evidence of the role of *MAB_1915* in drug resistance in *M. abscessus* by constructing a selectable marker-free in-frame deletion mutant for the gene (Fig. 1B and D) and complementing the knockout strain. Disruption of this gene was observed to significantly affect bacterial growth. A Tn insertion mutant for this gene has also been reported to show altered colony sizes and decreased growth in macrophages, suggesting that this gene could play an important role in survival of *M. abscessus* in macrophages (6, 22). In addition, the gene disruption has also been observed to render *M. abscessus* more susceptible to RIF, LZD, RFB, CLA, VAN, BDQ, CLF, and LEV (Table 1; Fig. 2). CP^Mab^, which expresses the CDS of *MAB_1915,* did not restore resistance to tested drugs, SDS, malachite green, and crystal violet, which is not surprising considering previous reports of little to no complementation of phenotypes involving FadDs (7, 10, 23, 24). However, intergenic genome regions could govern gene expression (25), and promoter regions could also be important determinants of drug resistance in mycobacteria (26). This led us to suspect a possible association between the role of *MAB_1915* in drug resistance and its upstream 272-bp region. We therefore constructed CP^UrMab^, a CP strain expressing a version of *MAB_1915* in which the CDS has been extended to include the 272-bp region. Interestingly, a remarkable restoration of resistance was observed in CP^UrMab^ (data not shown), suggesting that the role of *MAB_1915* in drug resistance could be dependent on this region. This observation could be explained by possible interruption (19, 27–29) and therefore a misannotation in the CDS of *MAB_1915*. We therefore shortened the length of *Ur-MAB_1915* (2,075 bp) to two versions *Psc1-MAB_1915* (1,872 bp) and *Psc2-MAB_1915* (1,830 bp) relative to the positions of two presumed “GTG” start codons (19) near the original annotated transcription start site (Fig. 3). CP strains expressing either of the shortened versions of *Ur-MAB_1915* have restored their drug resistance phenotypes (Table 1; Fig. 2). In addition, a bacterial promoter prediction program (BPROM) (30) identified “gggtaatct” as a potential promoter within this region, which lies some 94 bp and 136 bp upstream of *Psc1-MAB_1915* and *Psc2-MAB_1915,* respectively. Therefore, while the DST data clearly implicate *MAB_1915* in resistance to multiple drugs by *M. abscessus*, the hypothesized interruption in the CDS of *MAB_1915* and its relation to the promoter sequence remains to be studied.

FadDs play redundant (8) or independent roles such as synthesis of mycolic acids (16, 31) or specific lipids (10, 23, 24, 32, 33) of the mycobacterial cell envelope, which perhaps stems from their affinities for different chain lengths of fatty acids (2). We hypothesized therefore that increased drug susceptibility of the mutant strain could have resulted from altered cell envelope integrity, perhaps leading to increased permeability and easy passage of the drugs. This was tested by EtBr accumulation assay and susceptibility testing to SDS, malachite green, and crystal violet, which have been shown to be particularly bactericidal against or accumulate in bacteria with a compromised cell envelope (10, 34, 35). As expected, a higher accumulation of EtBr was observed in Mab^KO^ compared to Mab^Wt^, CP^Psc1,^ and CP^Psc2^ (*P* < 0.0001), a phenotype that was partially restored following complementation (Fig. 4A). In addition, increased susceptibility of the knockout strain to SDS, malachite green, and crystal violet was also observed to be partially reversed following complementation with extended versions of *MAB_1915* (Fig. 4B). Increased cell envelope permeability in Mab^KO^ could have resulted from defect in biosynthesis of α’-mycolates (6), which possibly caused some level of erosion of the mycobacterial cell envelope and paved the way for easier entry of drugs. α - and α’- mycolates are the two mycolic acid classes produced by *M. abscessus*, and disruption of *MAB_1915* abolishes the biosynthesis of the latter in *M. abscessus* ATCC 19977. The synthesis of the former class is mediated by FadD32 in *M. tuberculosis* (16), the *M. abscessus* ortholog for which is MAB_0179. In fact, structural comparison reveals that MAB_0179 differs from MAB_1915 only in its FadD32-specific insertion SI6 (6), suggesting that development of inhibitors for these two proteins could significantly inhibit the biosynthesis of mycolic acids in *M. abscessus* and therefore improve the effectiveness of chemotherapy against this pathogen. In the present study, however, thin layer chromatography of mycolate extracts did not indicate a change in the TLC profile of Mab^KO^ compared to Mab^Wt^ (data not shown). It is not clear whether this stems from strain variation between *M. abscessus* GZ002 (this study) and *M. abscessus* ATCC 19977 (6). For example, it is suspected that *MAB_1915* plays no role in the FASII pathway and therefore is not associated with mycolic acid biosynthesis because *MAB_1915* neither lies within the FASII operon of *M. abscessus* nor does it bear any orthology with the *fabD* gene (*Rv2243*) in the FASII operon in *M. tuberculosis* (36). This, however, appears to be speculative as evidence of such a role has been reported by Di Capua *et al*. (6). Nevertheless, it remains unclear whether this speculation is true for *M. abscessus* GZ002, and the potential effect of *MAB_1915* disruption on mycolate composition in this strain remains to be investigated. We ruled out the inhibition of lipid biosynthesis as a possible cause for increased cell envelope permeability of the mutant strain (6), which could be explained by various redundancies that abound lipid metabolism in mycobacteria, ranging from abundance of substrate-specific FadDs to alternative means of assimilation and metabolism of fatty acids (8, 37, 38).

The mycobacterial cell envelope represents a serious impediment to chemotherapy against mycobacteria, and drug targeting of its biogenesis could yield a great therapeutic outcome. Here, we highlighted the importance of *MAB_1915* to cell envelope permeability and resistance to multiple drugs in *M. abscessus*, hence presenting it as a novel target for development of effective antimicrobials to check the growing threat of this highly drug-resistant pathogen.

## MATERIALS AND METHODS

### Bacterial strains and growth conditions

Cloning experiments were carried out in *Escherichia coli* DH5α. *E. coli* was maintained in liquid or on solid Luria–Bertani medium under selective pressure (where needed) with the following concentrations of antibiotics (µg/mL): apramycin, APR 50; kanamycin, KAN 50; ampicillin, AMP 100; zeocin, ZEO 30. *M. abscessus* GZ002 (NCBI GenBank accession number CP034181) was maintained in Middlebrook 7H9 (Difco) medium supplemented with 0.2% glycerol, 0.05% Tween-80, and 10% OADC or on Middlebrook 7H10/7H11 supplemented with 0.5% glycerol and 10% OADC. *M. abscessus* GZ002 is a clinical strain with a smooth morphology from Guangzhou Chest Hospital, Guangzhou, Southern China (39). Selective growth of *M. abscessus* (when needed) was carried out in the presence of the following concentrations of antibiotics (µg/mL): KAN 100 or ZEO 30. All liquid cultures were maintained at 37°C under agitation.

### Preparation of competent *M. abscessus* cells

Electrocompetent cells were prepared as previously described (40–42) by washing three times in 10% glycerol. Briefly, pJV53-harboring *M. abscessus* cells were subcultured in Middlebrook 7H9 medium containing KAN and 0.2% succinic acid until the mid-log phase. Expression of the gp60/61 recombination proteins on pJV53 was induced by addition of 0.2% acetamide, and electrocompetent cells were prepared 5 hours later.

### Construction of *∆MAB_1915*

A three-fragment ligation method was used to construct an in-frame deletion of *MAB_1915* in *M. abscessus* by allelic exchange, as described previously (40). The AES was constructed by amplifying upstream (805 bp) and downstream (916 bp) arms of *MAB_1915* containing flanking sequences from adjacent genes. The amplified regions were cloned into *Cla*I/*Bam*HI-linearized pBlueSK(+) by recombination to obtain pBlueSK∆MAB_1915UD. The selective marker sandwiched between two *M. abscessus dif* sequences (*dif:zeoR:dif*, 600 bp) was also amplified by PCR. Both pBlueSK∆MAB_1915UD and *dif:zeoR:dif* were digested by *Hin*dIII, recovered, and ligated to construct pBlueSK∆MAB_1915UZD, the final plasmid containing the AES (*MAB_1915UZD*). *MAB_1915UZD* was amplified from the vector by PCR using the primer pair a/b (Fig. 1B). About 5 µg of purified AES was transformed into electrocompetent *M. abscessus*:pJV53 cells by electroporation (voltage, 2,500 V; capacitance, 25 µF; resistance, 1,000 Ω). Integration of the *zeoR* cassette into *M. abscessus* chromosome was verified by amplifying the chromosome*–zeoR* junction using the primer pairs a/c and d/b (Fig. 1B and C), and knockout was verified by PCR using the primer pair e/f (Fig. 1B and D) and sequencing.

### Construction of unmarked *∆MAB_1915* and CP strains

The *zeoR* cassette integrated into the *∆MAB_1915* chromosome after knockout was excised by multiple passages in the absence of selective pressure with the help of the Xer/*dif* system, as described previously (18). Each of several clones from passaged cells were grown on three separate plates, one containing no drug (plain), one containing ZEO, and the other containing KAN to ensure the shedding of pJV53. A colony that grows on the plain plate but not on the other two plates was selected and verified by PCR and sequencing. This colony was designated the unmarked mutant (Un1915) and was used as the background strain for construction of CP strains.

CP strains were constructed by amplifying either the annotated or extended CDS of *MAB_1915* and cloning into *Eco*RI/*Hin*dIII-linearized pMV261 and then transformed into Un1915 by electroporation. Construction of CP strains was verified by PCR and sequencing.

### DST

The bacterial strains were grown in 7H9 medium to an OD_600_ of 0.8–1. The resulting cultures were diluted in the same medium without Tween-80 to cell densities of ~10^5^ CFU/mL. Broth microdilution was used to determine MICs of selected drugs against the diluted cultures in transparent 96-well microplates. The MIC was defined as the lowest concentration at which visible growth was inhibited, and readings were recorded after 5 days of incubation at 37°C and after 14 days for CLA (43).

For DST using the SPOTi method, bacterial strains were grown in the 7H9 medium at 37℃ under agitation until OD_600_ 0.8–1 and then diluted to OD_600_ 0.6 in the same medium. The diluted cells or tenfold serial dilutions were spotted on plain and drug-containing Middlebrook 7H10/7H11 plates. Plates were observed after 5 days of incubation at 37℃. The MIC was defined as the lowest concentration at which at least 99% bacterial growth was inhibited.

### Ethidium bromide uptake assay

Cell envelope permeability was studied by EtBr accumulation assay (44–47) in FlexStation 3 Multi-Mode Microplate Reader (Molecular Devices, CA, USA) using the following parameters: temperature, 37°C; number of cycles and duration, 61 cycles of 60 seconds each; excitation and detection wavelengths, 530 nm and 595 nm, respectively. This was also studied by testing the bacterial susceptibility to two dyes (malachite green and crystal violet) and a detergent (SDS) by SPOTi (34). The concentration of EtBr used is 2 µg/mL, and susceptibility to dyes and detergents was tested on 7H11 plates and observed after 5 days of incubation at 37°C.

### Statistical analysis

Comparison of EtBr accumulation between strains was carried out by one-way analysis of variance with Tukey’s post-test to correct for multiple comparisons, at 95% CI and 0.05 significance level. Statistical analysis was carried out using GraphPad Prism version 8.0.2 (GraphPad, CA, USA), and *P* < 0.05 was considered significant.
